# Integrated Analysis of the Prognosis-Associated RNA-Binding Protein Genes and Candidate Drugs in Renal Papillary Cell Carcinoma

**DOI:** 10.3389/fgene.2021.627508

**Published:** 2021-02-12

**Authors:** Silin Jiang, Xiaohan Ren, Shouyong Liu, Zhongwen Lu, Aiming Xu, Chao Qin, Zengjun Wang

**Affiliations:** Department of Urology, The First Affiliated Hospital of Nanjing Medical University, Nanjing, China

**Keywords:** RNA-binding proteins, renal papillary cell carcinoma, prognostic model, bioinformatic, candidate drugs

## Abstract

RNA-binding proteins (RBPs) play significant roles in various cancer types. However, the functions of RBPs have not been clarified in renal papillary cell carcinoma (pRCC). In this study, we identified 31 downregulated and 89 upregulated differentially expressed RBPs on the basis of the cancer genome atlas (TCGA) database and performed functional enrichment analyses. Subsequently, through univariate Cox, random survival forest, and multivariate Cox regression analysis, six RBPs of SNRPN, RRS1, INTS8, RBPMS2, IGF2BP3, and PIH1D2 were screened out, and the prognostic model was then established. Further analyses revealed that the high-risk group had poor overall survival. The area under the curve values were 0.87 and 0.75 at 3 years and 0.78 and 0.69 at 5 years in the training set and test set, respectively. We then plotted a nomogram on the basis of the six RBPs and tumor stage with the substantiation in the TCGA cohort. Moreover, we selected two intersectant RBPs and evaluate their biological effects by GSEA and predicted three drugs, including STOCK1N-28457, pyrimethamine, and trapidil by using the Connectivity Map. Our research provided a novel insight into pRCC and improved the determination of prognosis and individualized therapeutic strategies.

## Introduction

Renal cell carcinoma (RCC), which accounts for 3% of adult malignancies, is a fatal malignancy of the urinary system (Huang et al., 2017). RCC consists of three subtypes: renal clear cell carcinoma (ccRCC), renal papillary cell carcinoma (pRCC), and renal chromophobe cell carcinoma (chRCC) (Tabibu et al., 2019). ccRCC constitutes 70% of all RCC cases, whereas pRCC is the second common subtype of RCC constituting 15% (Al Ahmad et al., 2019). Clinically, pRCC is considered as more inert than ccRCC. However, advanced cases of pRCC have metastatic potential, which are more lethal than ccRCC (Kaldany et al., 2019). Therefore, a comprehensive analysis of vital genes in pRCC tumorigenesis is typically necessary to evaluate the individual prognosis, determine the therapeutic target, and predict potential drugs for patients with poor prognosis.

RNA-binding proteins (RBPs) are a significant group of cellular proteins containing RNA-binding domains, which play a key role in the post-transcriptional regulation of gene expression, such as RNA shearing, transport, stability, protein translation, and subcellular localization (Burd and Dreyfuss, 1994; Glisovic et al., 2008). Recent studies have revealed links between RBPs and known cancer biomarkers (Kechavarzi and Janga, 2014; Pereira et al., 2017). For example, high-risk HPV E7 activates RBP RNASEH2A and PCNA expression, and PCNA directs RNASEH2A activity with regard to DNA replication. The induction of these two factors may promote DNA replication and cancer cell proliferation (Xu et al., 2019). High expression of RBP LARP1 co-associates with BCL2 and BIK in BCL2 messenger ribonucleoprotein (mRNP) complexes in epithelial ovarian cancer and stabilizes BCL2 while destabilizing BIK, which promotes ovarian cancer cell survival and leads to adverse prognosis (Hopkins et al., 2016). RBP NELFE decreases the stabilization of NDRG2 mRNA, which results in epithelial-to-mesenchymal transition through the activation of the Wnt/β-catenin signaling and promotion of the metastasis of pancreatic cancer (Han et al., 2019). Thus, defects or dysfunctional RBP is bound with tumorigenesis and tumor prognosis.

As the high-throughput sequencing platforms develop, the vast amount of genomic data are applied for biomarker prediction, prognosis analysis, and targeted therapy (Azad and Li, 2013; Gounder et al., 2015). Bioinformatic analyses provide abundant tools and specific algorithms to obtain, process, and interpret biological data (Psarros et al., 2005). In this study, we conducted a series of bioinformatic analyses on the basis of the biological data downloaded from the cancer genome atlas (TCGA) database and finally sifted six differentially expressed RBPs associated with pRCC. Our results might provide a new direction for the understanding of progression and prognosis of pRCC.

## Materials and Methods

### Data Acquisition

The FPKM transcriptome profiling data of 32 normal samples and 289 pRCC samples were obtained from the TCGA database (Wang et al., 2016). Clinical data were downloaded from the TCGA database. A total of 1542 RBP genes were obtained from the published literature (Gerstberger et al., 2014).

### Data Processing of Differentially Expressed Genes (DEGs)

We used limma package in R software to identify differentially expressed genes (DEGs) of RBPs between the tumor and normal groups. The identification was based on cutoffs of |log2 fold change (FC)| > 0.5 and false discovery rate (FDR) < 0.05.

### Functional Enrichment Analyses and Protein–Protein Interaction Network

Gene ontology (GO), which contained three terms (biological process [BP], cellular component [CC], and molecular function [MF]), was applied to investigate the biological function enrichment. The Kyoto Encyclopedia of Genes and Genomes database (KEGG) was applied to identify the potential biological pathways. All GO and KEGG enrichment analyses were conducted on R software through the clusterProfiler R package with a *P* value less than 0.05. The protein--protein interactions (PPIs) among DEGs were checked using the Search Tool for the Retrieval of Interacting Genes (STRING) database^1^ (Gao et al., 2013). Cytoscape 3.7.2 (Doncheva et al., 2019) was used for the visualization of the PPI network. Subsequently, the molecular complex detection (MCODE) plug-in in Cytoscape was loaded to filter out significant modules from the PPI network with score >5 and node counts >5.

### Prognostic Model Construction and Analyses

After establishing a combination by merging gene expression and overall survival (OS), we conducted univariate Cox regression to select prognosis-related RBP genes (*P* < 0.01). We applied randomForestSRC package in R software to conduct the random survival forest-variable hunting algorithm to predict the significant RBP genes from initially screened candidates. On the basis of these genes, a prognostic model was constructed by multivariate Cox regression, and the rick score was calculated according to the following formula:

Risk Score $\sum_{i=1}^{N}\alpha_{i}\,x_{i}$

N represents the number of selected genes; α is the coefficient of genes in the Cox regression analysis, and *x* indicates the gene expression value. *P* values computed by Kaplan–Meier (KM) analysis were then sorted to sift the best combination of six genes. pRCC patients from the TCGA database were divided randomly into the training set and test set by using createDataPartition function in caret R package, and patients in either set were further categorized into the high-risk group and low-risk group according to their median risk score. The survival R package and pROC R package were conducted to construct a receiver operating characteristic (ROC) curve and measure the accuracy of prognosis. In addition, we plotted a nomogram to calculate the feasibility of OS using the nomogramEx R package.

### Gene Set Enrichment Analysis

Considering that SNRPN and RRS1 were intersections between critical module 1 of the PPI network and prognosis-related combination, gene set enrichment analysis (GSEA) v4.1.0 was downloaded from Broad Institute, and Hallmark gene set V7.2 collection was downloaded as the target set to analyze the potential mechanism of actions of two genes.

### Prediction of Candidate Small-Molecule Drugs

We identified differentially expressed RBPs of the high and low-risk groups by applying the limma R package. Then, the Connectivity Map^2^ was used to predict small molecules as potential targeted drugs for pRCC (Lamb et al., 2006).

### Statistical Analysis

We used the Perl language^3^ to merge the transcriptome and clinical data. All statistical analyses were performed in Cytoscape 3.7.2 (Doncheva et al., 2019), GSEA v4.1.0 (Subramanian et al., 2005) and R version 3.6.3 with the following packages: “limma” (Ritchie et al., 2015), “clusterProfiler” (Yu et al., 2012), “survival,” “randomForestSRC,” “caret,” “pROC,” “nomogramEX.”

## Results

### Exploration of DEGs

**FIGURE 1:**
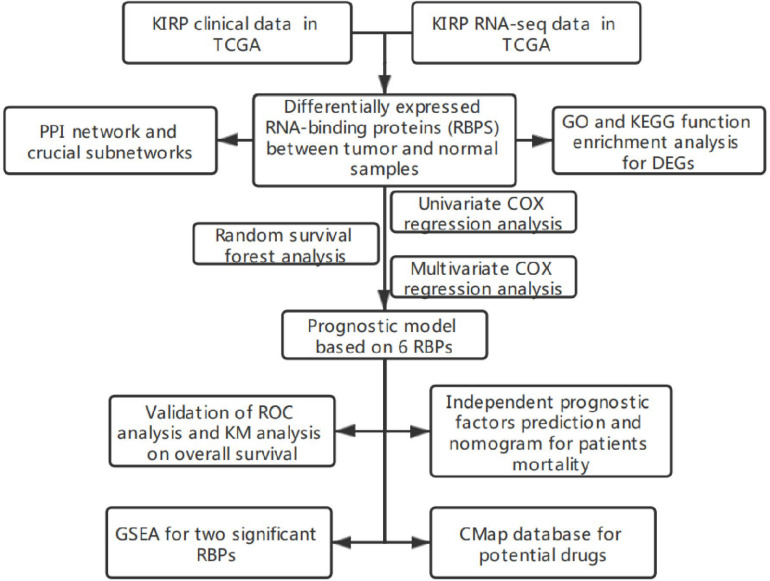
Flowchart of analyzation on RBPs in renal papillary cell carcinoma (pRCC).

**FIGURE 2:**
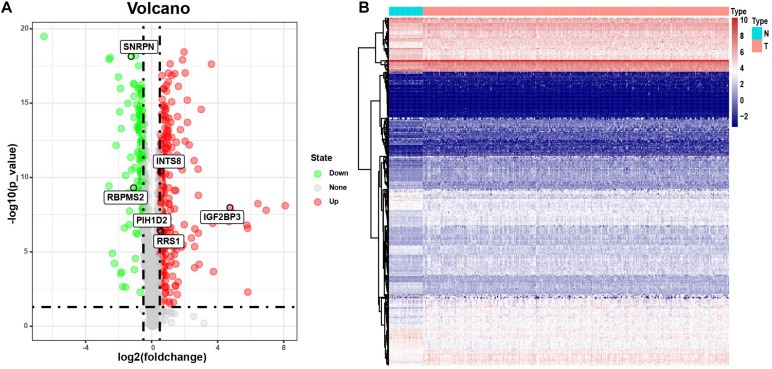
The differentially expressed RBPs. **(A)** Volcano plot of differentially expressed RBPs reaching the threshold of adj. *P* < 0.05 and | log2FC| ≥ 0.5. **(B)** Heatmap plot of 120 differentially RBPs expression between tumor and normal samples in pRCC.

The flowchart of this study is illustrated in Figure 1. RNA sequencing data containing 32 normal samples and 289 tumor samples of pRCC and clinical data were downloaded from the TCGA database. The list of 1542 RBP genes was acquired from published literature (Gerstberger et al., 2014). Finally, 380 RBP-coding genes containing 129 downregulated and 251 upregulated genes met our inclusion criteria of adj. *P* < 0.05 and |log2FC| ≥0.5 (Supplementary Table 1). The expression of DEGs was plotted in a heatmap for visualization (Figures 2A,B). 285 tumor samples were selected after filtering out samples which were lack of clinical survival information.

### Functional Enrichment Analyses of DEGs

**FIGURE 3:**
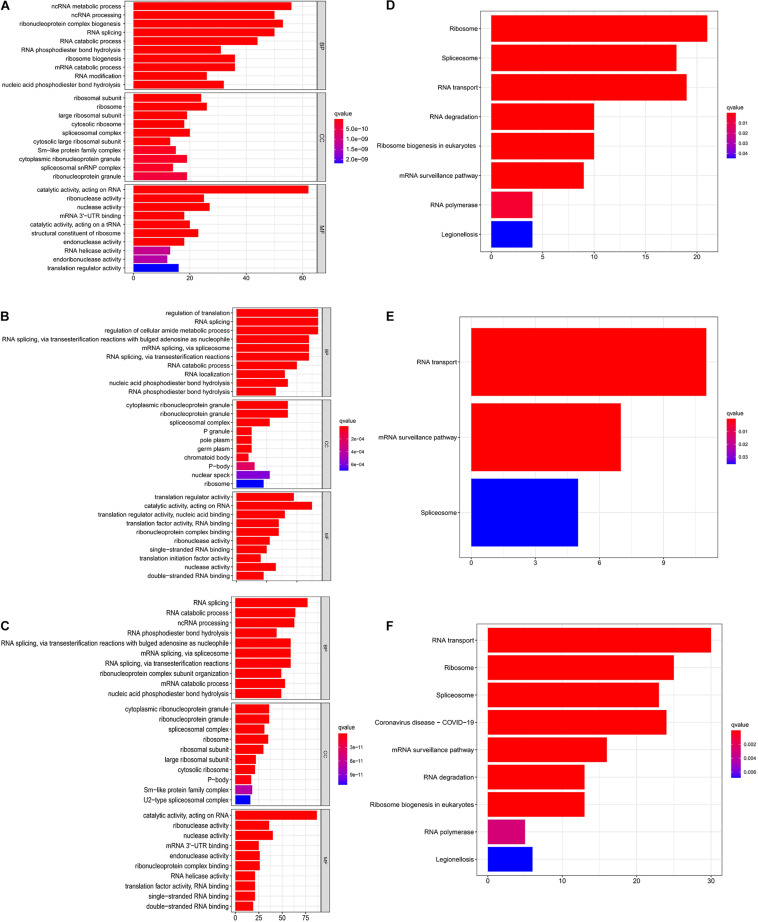
Functional enrichment analyses of 251 upregulated and 129 downregulated RBPs. **(A)** GO analysis on upregulated RBPs. **(B)** GO analysis on downregulated RBPs. **(C)** GO analysis on all the DERBPs. **(D)** KEGG analysis on upregulated RBPs. **(E)** KEGG analysis on downregulated RBPs. **(F)** KEGG analysis on all the DERBPs.

Gene ontology and KEGG analyses were conducted on downregulated and upregulated genes using the clusterProfiler R package. The upregulated RBPs were conspicuously enriched in BPs, including ncRNA metabolic process, ncRNA processing, ribonucleoprotein complex biogenesis, and RNA splicing, whereas the downregulated RBPs were enriched in the regulation of translation, RNA splicing, and regulation of the cellular amide metabolic process. CC analyses demonstrated that the upregulated RBPs were notably enriched in the ribosomal subunit, ribosome, spliceosomal complex, and cytosolic ribosome, whereas the downregulated RBPs were enriched in the cytoplasmic ribonucleoprotein granule, ribonucleoprotein granule, and spliceosomal complex. With regard to MF analyses, the results indicated that the upregulated RBPs were significantly enriched in the catalytic activity, acting on RNA, ribonuclease activity, nuclease activity, and mRNA 3′–UTR binding, and the downregulated RBPs were enriched in the translation regulator activity and catalytic activity, acting on RNA, translation regulator activity, nucleic acid binding, translation factor activity, and RNA binding (Figures 3A,B). As for the KEGG analyses, we discovered that the upregulated RBPs were largely enriched in the pathways of the ribosome, spliceosome, and RNA transport (Figure 3D). The downregulated RBPs were primarily enriched in the RNA transport pathway and mRNA surveillance pathway (Figure 3E). Furthermore, we performed GO and KEGG analyses on all the DERBPs in order to have a more scientifically comprehensive understanding. The GO results revealed that DEGs were mainly enriched in RNA splicing and RNA catabolic process in BP analysis, cytoplasmic ribonucleoprotein granule and ribonucleoprotein granule in CC analysis, as well as catalytic activity and acting on RNA, nuclease activity in MF analysis (Figure 3C). According to KEGG analysis, DEGs were significantly enriched in RNA transport, RNA transport and Coronavirus disease–COVID-19 (Figure 3F).

### PPI Network Construction and Crucial Module

**FIGURE 4:**
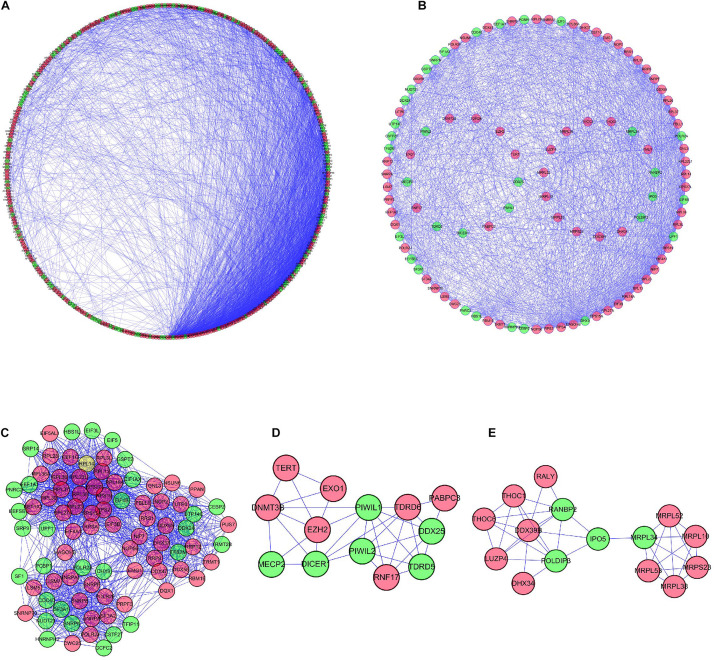
PPI network based on 380 differentially expressed RBPs and critical subnetworks. **(A)** Visualization of PPI network conducted on Cytoscape. Red nodes represent upregulated RBPs and green nodes represent downregulated RBPs. **(B)** Visualization of three MCODE modules. Visualization of module 1 **(C)**, module 2 **(D)**, and module 3 **(E)**.

The PPI network was constructed by using the STRING database and visualized by applying Cytoscape (Figure 4A). This PPI network comprised 346 nodes and 3164 edges. Furthermore, we extracted three modules using plug-in MCODE in Cytoscape according to the cutoff of node counts >5 and score >5 (Figure 4B). Module 1 consisted of 85 nodes and 1366 edges; module 2 comprised 13 nodes and 33 edges, and module 3 included 15 nodes and 38 edges (Figures 4C–E). The results of GO and KEGG for three modules indicated that the genes in module 1 were primarily enriched in the ribonucleoprotein complex biogenesis, cytosolic ribosome, structural constituent of ribosome, and ribosome pathway. The genes in module 2 were enriched in DNA alkylation, chromatoid body, regulatory RNA binding, and MicroRNAs in cancer, whereas the genes in module 3 were significantly enriched in the mitochondrial translational elongation, organellar large ribosomal subunit, structural constituent of ribosome, and RNA transport pathway (Supplementary Tables 2, 3).

### Prognostic Risk Score Model Construction and Validation

**FIGURE 5:**
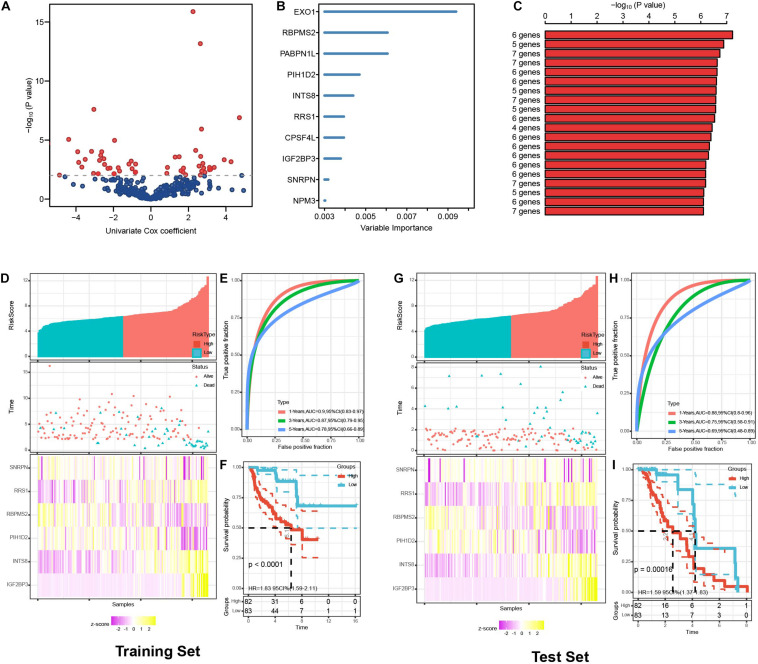
Identification of prognosis-related RBPs and validations of prognostic risk score model in training set and test set. **(A)** Volcano plot represented the prognosis-related RBPs of univariate Cox regression analysis. **(B)** Random survival forest analysis filtered out 10 best related genes. **(C)** The top 20 signatures were screened out among 1023 combinations according to the *P* value of Kaplan–Meier analysis. The risk score distribution, survival status distribution and heatmap of six RBPs expression in the training set **(D)** and test set **(G)**. ROC analysis for predictive OS of pRCC patients at 1, 3, and 5 years in the training set **(E)** and test set **(H)**. Kaplan–Meier analysis for overall survival (OS) of different risk groups in the training set **(F)** and test set **(I)**.

A combination of 346 RBPs from the PPI network and OS was analyzed through univariate Cox regression to confirm prognosis-related RBPs (Figure 5A). Sixty RBPs were sorted out by the cutoff of *P* value < 0.01. The randomForestSRC R package was applied to perform Random survival forest analysis, thereby distinguishing the RBP genes with the best association with prognosis. In addition, 10 genes (EXO1, RBPMS2, PABPN1L, PIH1D2, INTS8, RRS1, CPSF4L, IGF2BP3, SNRPN, and NPM3) were screened out from the 60 prognosis-related RBPs (Figure 5B). Subsequently, multivariate Cox analysis was conducted to establish a prognostic model related to OS, and we further performed a KM analysis on the 2^10^ = 1023 models formed by 10 genes to determine the best risk score model (Figure 5C). Comparing the *log*_*10*_−l rank *P* value of these 1023 models, we finally sorted out the prognostic risk score model containing six RBPs (SNRPN, RRS1, INTS8, RBPMS2, IGF2BP3, and PIH1D2). The risk score of each pRCC patient was calculated as follows: Risk score = (0.2729931 × SNRPN) + (0.9340297 × RRS1) + (1.8014324 × INTS8) + (−0.5129049 × RBPMS2) + (1.7546410 × IGF2BP3) + (−0.4098881 × PIH1D2).

We allocated pRCC patients into the training and test sets to evaluate the predictive capabilities of the model, and then patients in each set were divided into the high- and low-risk groups considering their median risk score (Table 1). Expressions of survival status and heatmap of each set were also shown (Figures 5D,G). ROC analyses were utilized to estimate the prognostic model. The area under the curve (AUC) values in the training set were 0.9 at 1 year, 0.87 at 3 years, and 0.78 at 5 years, whereas the AUC values in the test set were 0.88 at 1 year, 0.75 at 3 years, and 0.69 at 5 years (Figures 5E,H). The results indicated that patients in the high-risk group showed a significantly lower survival probability than those in the low-risk group (Figures 5F,I).

**TABLE 1:** Clinical features of pRCC patients in the training and test set.

Features	Subgroups	All (*n* = 285)	Training set (*n* = 143)	Test set (*n* = 142)
Age	<60	118	62	56
	≥60	165	81	84
	NA	2	0	2
Gender	Male	209	100	109
	Female	76	43	33
Pathological Stage	I	170	67	103
	II	21	13	8
	III	50	29	21
	IV	15	9	6
	NA	29	25	4
T Staging	T1	191	84	107
	T2	32	21	11
	T3	58	38	20
	T4	2	0	2
	NA	2	0	2
N Staging	N0	49	30	19
	N1	23	10	13
	N2	4	2	2
	NA	209	101	108
M Staging	M0	95	55	40
	M1	9	7	2
	NA	181	81	100

**FIGURE 6:**
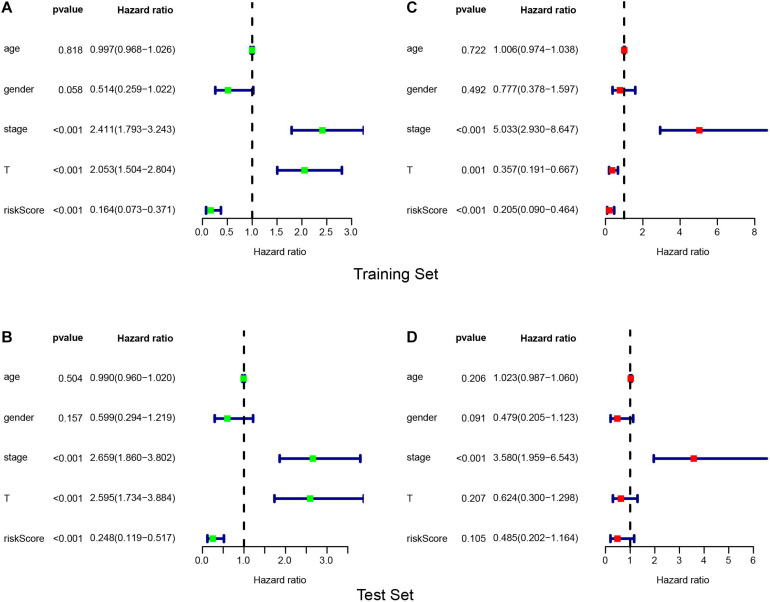
Identification of independent prognostic factors. Outcomes of univariate prognostic analysis conducted on training set **(A)** and test set **(B)**. Outcomes of multivariate prognostic analysis conducted on training set **(C)** and test set **(D)**.

**FIGURE 7:**
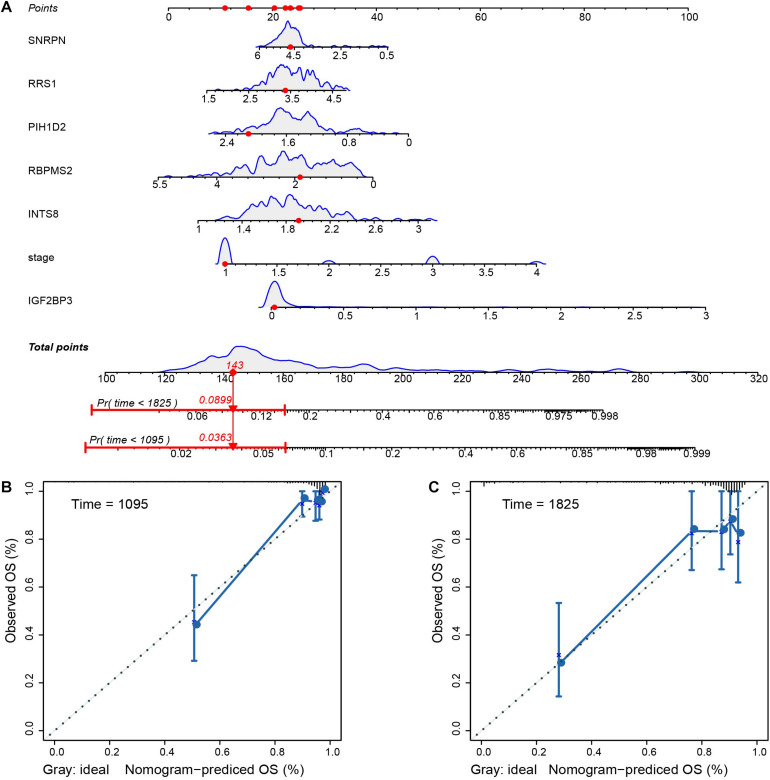
Nomogram for predicting the probability of patient mortality based on six RBPs as well as tumor stage, and calibrations of nomogram in terms of conformity between predicted outcomes and observed outcomes at 3 and 5 years. **(A)** Nomogram for evaluating the possibility of pRCC patients mortality at 3 and 5 years. Calibration for assessing the conformity between nomogram OS and observed OS at 3 years **(B)** and 5 years **(C)**.

We conducted an independent prognosis-related analysis on the training and test sets by using univariate and multivariate COX regression analyses to appraise the clinical factors in prognosis. The univariate results indicated that in the training and test sets, the stage and T staging could be considered as independent prognostic factors for the OS of pRCC patients (Figures 6A,B). In multivariate analysis, tumor stage and T staging could be considered as independent prognostic factors in the training set (Figure 6C). Nevertheless, only tumor stage can be considered as an independent prognostic factor in the test set (Figure 6D). Finally, the nomogram was constructed with six selected genes and tumor stage to evaluate the mortality risk at 3 and 5 years (Figure 7A). Furthermore, we plotted the calibration curves, which demonstrated the ideal conformity between speculated outcomes and observed outcomes (Figures 7B,C).

### Gene Set Enrichment Analysis

**FIGURE 8:**
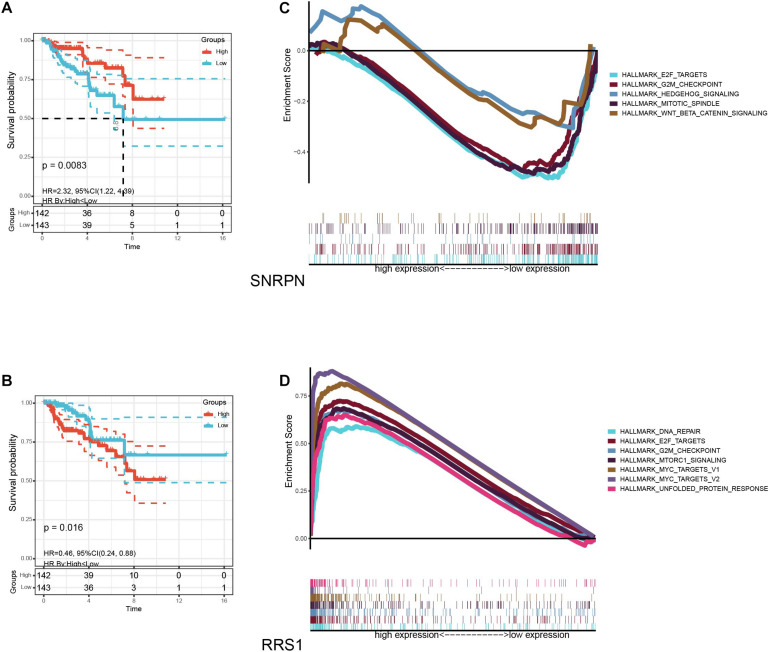
The survival curves and GSEA for samples with high and low expression of SNRPN and RRS1. **(A)** Kaplan–Meier analysis for overall survival (OS) based on the expression of SNRPN. **(B)** Kaplan–Meier analysis for overall survival (OS) based on the expression of RRS1. **(C)** The enriched gene sets in HALLMARK collection by samples with low SNRPN expression. **(D)** The enriched gene sets in HALLMARK collection by samples with high RRS1 expression.

We found that SNRPN and RRS1 were intersections between the prognostic model and module 1 of the PPI network and then made a KM analysis for OS (Figures 8A,B). Given that the levels of SNRPN were negatively correlated with survival, whereas RRS1 was positively correlated with survival, GSEA analysis was applied in the low-expression and high-expression groups. In the SNRPN low-expression group, the gene sets were enriched in mitotic spindle, E2F targets, and G2M checkpoints (Figure 8C). On the contrary, in the RRS1 high-expression group, the genes sets in hallmark collection were enriched in DNA repair, E2F targets, G2M checkpoints, MTORC1 signaling, MYC targets, and unfolded protein response (Figure 8D).

### Screening of Candidate Small-Molecule Drugs

We applied the limma R package to identify the differential expression of RBPs in different risk groups. Consequently, 297 RBPs consisting of 261 upregulated and 36 downregulated RBPs reached the threshold of adj. *P* < 0.05 and |log_2_FC| > 1. The prediction of small-molecule drugs was based on the 297 RBPs. Finally, three small molecules (STOCK1N-28457, pyrimethamine, and trapidil) were selected on the basis of the enrichment score (>0.6), *P* value (<0.05), and percent non-null (>70, Table 2).

**TABLE 2:** Results of CMap analysis.

Cmap name	Mean	*n*	Enrichment	*p*	Specificity	Percent non-null
STOCK1N-28457	–0.717	3	–0.93	0.00052	0.0051	100
Pyrimethamine	–0.678	5	–0.773	0.00108	0.0067	80
Trapidil	–0.58	3	–0.889	0.00266	0.0124	100
AH-6809	0.679	2	0.934	0.00833	0	100
Mercaptopurine	–0.61	2	–0.908	0.01714	0.0137	100
7-aminocephalosporanic acid	–0.389	4	–0.699	0.01717	0.0311	75
Lobelanidine	0.477	4	0.689	0.01936	0.0067	75
5230742	–0.648	2	–0.897	0.0209	0.0383	100
Emetine	–0.42	4	–0.677	0.02401	0.2765	75
Spaglumic acid	0.598	2	0.891	0.02463	0.0397	100
Exisulind	0.524	2	0.891	0.02487	0	100
Loxapine	0.47	4	0.667	0.02751	0.0106	75
Debrisoquine	0.441	4	0.64	0.041	0.0204	75
Altizide	0.413	4	0.639	0.04132	0	75
Oxybenzone	0.431	4	0.634	0.04416	0.1706	75

## Discussion

Recently, RBPs were becoming important by the profound study conducted on its roles in various cancers and increasingly regarded as crucial factors in post-transcriptional regulation (Dong et al., 2019; Singh et al., 2018; Soni et al., 2019). The dysfunction of post-transcriptional regulation, which was related to the origin of cancer, were associated with gain−of−function mutations of oncogenes and loss−of−function mutations of the tumor suppressor (Masuda and Kuwano, 2019; Vogelstein and Kinzler, 2015). To the best of our knowledge, this study focused on the role of RBPs in the progression and prognosis of pRCC for the first time. Here, we integrated RNA sequencing data of pRCC from the TCGA database and sorted out differentially expressed RBPs between tumor and normal samples. We further conducted GO and KEGG enrichment analyses and established the PPI network for these RBPs. Moreover, we constructed an OS-predictive model to predict the prognosis of pRCC patients and performed ROC analyses to evaluate the feasibility of our model. Subsequently, GSEA was conducted to determine the biological functions of the two selected RBPs.

As for the results of biological functions and pathway enrichment analyses, DEGs were enriched in the ribosome and post-transcriptional modification pathways, such as RNA splicing, RNA transport, spliceosome, and translation. Several studies in recent years have reported that aberrant RNA modification and RNA metabolism were of great value in various cancers (Delaunay and Frye, 2019; Li et al., 2017b). Li et al. (2019) reported that alternative RNA splicing events, which were probably adjusted by RBPs, were prevalent in liver cancer affecting tumorigenesis in the metabolism-related pathways. In addition, the manipulation of alternative splicing was proven to be a new method to suppress tumorigenesis in glioblastoma by Mogilevsky et al. (2018). Moreover, Will discovered that spliceosome consisted of five snRNPs and numerous proteins, which catalyzed pre-mRNA splicing (Will and Lührmann, 2011). The variable levels of RNA and protein components affected the splice site, and a research conducted by Dvinge et al. (2019) showed that the spliceosome shaped the global transcriptome of breast cancer. In ovarian cancers, Li et al. (2017a) found that spliceosome could promote proliferation and invasion by the upregulation of an associate factor. Previous studies have shown the relationship and possible mechanisms between RBPs and spliceosome (Naro and Sette, 2013; Sutandy et al., 2018).

Subsequently, through the application of univariate Cox regression analysis, random survival forest analysis, multivariate Cox analysis, and KM test, we determine six RBP-coding genes: SNRPN, RRS1, INTS8, RBPMS2, IGF2BP3, and PIH1D2. The risk score model was then constructed to predict the prognosis of patients. Notably, patients with high risk scores had worse prognosis, implying that individual therapeutic schedules should be considered. The ROC curve of the risk score model revealed that the six-RBP signature was comparatively reliable in predicting prognosis with the AUC values of 0.87 and 0.75 at 3 years and 0.78 and 0.69 at 5 years in the training and test sets, respectively. A nomogram comprised an independent prognostic factor, and six-RBP signature was established to assist the prediction of 3 and 5 year OS in clinical treatments.

In addition, we performed GSEA on SNRPN and RRS1 because they were concurrently parts of the subnetwork of PPI. Small nuclear ribonucleoprotein polypeptide N (SNRPN) was widely regarded as a spliceosome component (Jing et al., 2015). As shown in the results of GSEA, low-expression SNRPN was enriched in the E2F targets. E2F was a family of transcription factors, which had various functions such as controlling the cell cycle, regulating transcription, and apoptosis (Johnson and Schneider-Broussard, 1998). Moreover, the E2F targets played significant roles in several cancers. For example, Park et al. (2018) announced that the E2F targets were activated by EPEL to promote cell proliferation of lung cancer. Meanwhile, Sun et al. (2019) researched on the roles of E2Fs in breast cancer and considered E2F4 and 6 as biomarkers, with E2F1, 3, 5, 7, and 8 as potential targets of therapy. Dong et al. (2018) reported that the inhibition of E2F downregulated the ability of BRD4 binding with the promoter of miR-106b-5p and inhibited its transcription, which resulted in the cellular senescence of gastric cancer cells (Sutandy et al., 2018). High-expression RRS1 was enriched in mTORC1 signaling. The mTORC1 signaling pathway was a classical pathway connecting to tumorigenesis (Dong et al., 2018). He et al. (2018) and Guigon et al. (2010) reported that the growth of pancreatic cancer and thyroid cancer were inhibited by the suppression of mTORC1 signaling (Poburski et al., 2016). Experiments on the interaction mechanism of TRAF6 and p62 were carried out by Linares et al. (2013). The results indicated the importance for lung cancer cell proliferation through the activation of mTORC1 (Guigon et al., 2010). Furthermore, we made a prediction of potential small-molecule drugs, which might be of therapeutic benefits for pRCC patients and had a certain degree of reliability.

Our study investigated the relationship between RBPs and pRCC for the first time and proposed a novel direction for exploring the tumorigenesis and prognosis of pRCC. We determined six RBPs, which were linked with prognosis, constructed a reliable prognostic OS-predictive model, and hypothesized three potentially useful drugs. The six RBPs could act as potential therapeutic targets of pRCC and contribute to the development of clinical treatment. Nevertheless, our study had several limitations. First, our prognostic model was only constructed on the TCGA database, which lacked of clinical data from the GEO database to evaluate. Meanwhile, the lack of clinical characteristics of clinical data from TCGA might decrease the credibility of our research. Moreover, our results were based on RNA sequencing, and patients might exhibit inter-individual heterogeneity. Finally, prospective clinical studies should be conducted before using the six-RBP prognostic model.

## Conclusion

We applied a series of bioinformatic analyses on the aberrantly expressed RBPs, which were affiliated with tumorigenesis, invasion, and prognosis, to investigate their potential functions, action pathways, and prognostic values. Subsequently, we identified six RBPs, which were highly associated with prognosis of pRCC, and constructed a six-RBP prognostic model to predict the OS and optimize the predictive ability of the staging system. Moreover, we selected two important RBPs and evaluated their biological effects and made a prediction of potential drugs. Based on previous reports, this study focused on the prognostic values of RBPs in pRCC and provided new insights into pathogenesis and therapeutic strategies of pRCC for the first time.

## Data Availability Statement

The datasets presented in this study can be found in online repositories. The names of the repository/repositories and accession number(s) can be found in the article/Supplementary Material.

## Author Contributions

ZW: conception and design of the study and funding acquisition. SJ and XR: data acquisition, bioinformatics analysis, and drafting and critical revision of the manuscript. SL and ZL: visualization and validation. All authors approved the final manuscript.

## Conflict of Interest

The authors declare that the research was conducted in the absence of any commercial or financial relationships that could be construed as a potential conflict of interest.

## Footnotes

1 https://string-db.org/

2 https://portals.broadinstitute.org/cmap/

3 https://www.perl.org/

## Supplementary Material

Supplementary Table 1 The gene expression differences for each gene.

Supplementary Table 2 The GO functional enrichment of three crucial modules.

Supplementary Table 3 The KEGG functional enrichment analysis of threecrucial modules.

## Abbreviations

pRCC: renal papillary cell carcinoma
RBP: RBA-binding protein
DEGs: differentially expressed genes
GO: Gene Ontology
KEGG: the Kyoto Encyclopedia of Genes and Genomes Database
PPI: protein–protein interaction
FC: fold change
FDR: false discovery rate
ROC: receiver operating characteristic
AUC: area under the curves
GSEA: gene set enrichment analysis
OS: overall survival
TCGA: the cancer genome atlas.
