# Supplementary material for: Integrated Analysis of the Prognosis-Associated RNA-Binding Protein Genes and Candidate Drugs in Renal Papillary Cell Carcinoma
Source: Front Genet. 2021 Feb 12;12:627508. doi: 10.3389/fgene.2021.627508 (PMC7907657; doi:10.3389/fgene.2021.627508)
Supplement: Supplementary Table 2 — The GO functional enrichment of three crucial modules. [file Table_2.doc]

**Table 2. The GO functional enrichment of three crucial modules.**

| **ONTOLOGY** | **ID** | **Description** | **pvalue** | **p.adjust** | **Count** |
| --- | --- | --- | --- | --- | --- |
| Module 1 | | | | | |
| BP | GO:0022613 | ribonucleoprotein complex biogenesis | 2.13E-33 | 1.09E-30 | 33 |
| BP | GO:0000956 | nuclear-transcribed mRNA catabolic process | 6.85E-26 | 1.38E-23 | 22 |
| BP | GO:0000184 | nuclear-transcribed mRNA catabolic process, nonsense-mediated decay | 8.14E-26 | 1.38E-23 | 19 |
| CC | GO:0022626 | cytosolic ribosome | 8.69E-25 | 8.60E-23 | 18 |
| CC | GO:0044391 | ribosomal subunit | 1.74E-20 | 8.63E-19 | 18 |
| CC | GO:0022625 | cytosolic large ribosomal subunit | 1.27E-19 | 4.18E-18 | 13 |
| MF | GO:0003735 | structural constituent of ribosome | 2.72E-19 | 3.02E-17 | 18 |
| MF | GO:0090079 | translation regulator activity, nucleic acid binding | 8.11E-16 | 4.50E-14 | 13 |
| MF | GO:0008135 | translation factor activity, RNA binding | 1.38E-15 | 5.10E-14 | 12 |
| Module 2 | | | | | |
| BP | GO:0006305 | DNA alkylation | 9.31E-10 | 2.84E-07 | 5 |
| BP | GO:0006306 | DNA methylation | 9.31E-10 | 2.84E-07 | 5 |
| BP | GO:0044728 | DNA methylation or demethylation | 3.62E-09 | 7.38E-07 | 5 |
| CC | GO:0033391 | chromatoid body | 6.66E-14 | 2.46E-12 | 5 |
| CC | GO:0036464 | cytoplasmic ribonucleoprotein granule | 2.32E-09 | 3.88E-08 | 6 |
| CC | GO:0035770 | ribonucleoprotein granule | 3.14E-09 | 3.88E-08 | 6 |
| MF | GO:0061980 | regulatory RNA binding | 9.85E-09 | 6.01E-07 | 4 |
| MF | GO:0140098 | catalytic activity, acting on RNA | 4.37E-08 | 1.02E-06 | 6 |
| MF | GO:0004521 | endoribonuclease activity | 5.04E-08 | 1.02E-06 | 4 |
| Module 3 | | | | | |
| BP | GO:0070125 | mitochondrial translational elongation | 4.46E-11 | 7.24E-09 | 6 |
| BP | GO:0070126 | mitochondrial translational termination | 4.78E-11 | 7.24E-09 | 6 |
| BP | GO:0006415 | translational termination | 1.24E-10 | 1.25E-08 | 6 |
| CC | GO:0000315 | organellar large ribosomal subunit | 4.96E-10 | 9.17E-09 | 5 |
| CC | GO:0005762 | mitochondrial large ribosomal subunit | 4.96E-10 | 9.17E-09 | 5 |
| CC | GO:0000313 | organellar ribosome | 4.32E-09 | 3.99E-08 | 5 |
| MF | GO:0003735 | structural constituent of ribosome | 1.51E-05 | 0.000377 | 4 |
| MF | GO:0008536 | Ran GTPase binding | 0.000424 | 0.005294 | 2 |
| MF | GO:0003724 | RNA helicase activity | 0.001686 | 0.014054 | 2 |
