# Supplementary material for: Integrated Analysis of the Prognosis-Associated RNA-Binding Protein Genes and Candidate Drugs in Renal Papillary Cell Carcinoma
Source: Front Genet. 2021 Feb 12;12:627508. doi: 10.3389/fgene.2021.627508 (PMC7907657; doi:10.3389/fgene.2021.627508)
Supplement: Supplementary Table 3 — The KEGG functional enrichment analysis of threecrucial modules. [file Table_3.doc]

**Table 3. The KEGG functional enrichment analysis of three crucial modules.**

| **ID** | **Description** | **pvalue** | **p.adjust** | **Count** |
| --- | --- | --- | --- | --- |
| Module 1 | | | | |
| hsa03010 | Ribosome | 2.38E-18 | 4.04E-17 | 18 |
| hsa03040 | Spliceosome | 8.99E-12 | 7.64E-11 | 13 |
| hsa03013 | RNA transport | 2.78E-06 | 1.58E-05 | 9 |
| hsa03015 | mRNA surveillance pathway | 3.74E-05 | 0.000159 | 6 |
| hsa03008 | Ribosome biogenesis in eukaryotes | 8.00E-05 | 0.000272 | 6 |
| hsa05134 | Legionellosis | 0.000505 | 0.001431 | 4 |
| hsa03020 | RNA polymerase | 0.001054 | 0.00256 | 3 |
| Module 2 | | | | |
| hsa05206 | MicroRNAs in cancer | 0.001028 | 0.011308 | 3 |
| Module 3 | | | | |
| hsa03013 | RNA transport | 3.94E-06 | 1.97E-05 | 4 |
| hsa03040 | Spliceosome | 0.00496 | 0.009032 | 2 |
| hsa03010 | Ribosome | 0.005419 | 0.009032 | 2 |
